# Supplementary material for: Structural insights on TRPV5 gating by endogenous modulators
Source: Nat Commun. 2018 Oct 10;9:4198. doi: 10.1038/s41467-018-06753-6 (PMC6179994; doi:10.1038/s41467-018-06753-6)
Supplement: Supplementary file 1 — Supplementary Information [file 41467_2018_6753_MOESM1_ESM.pdf]

Structural insights on TRPV5 gating by endogenous modulators

Taylor E.T. Hughes, Ruth A. Pumroy, Aysenur Torun Yazici et al.

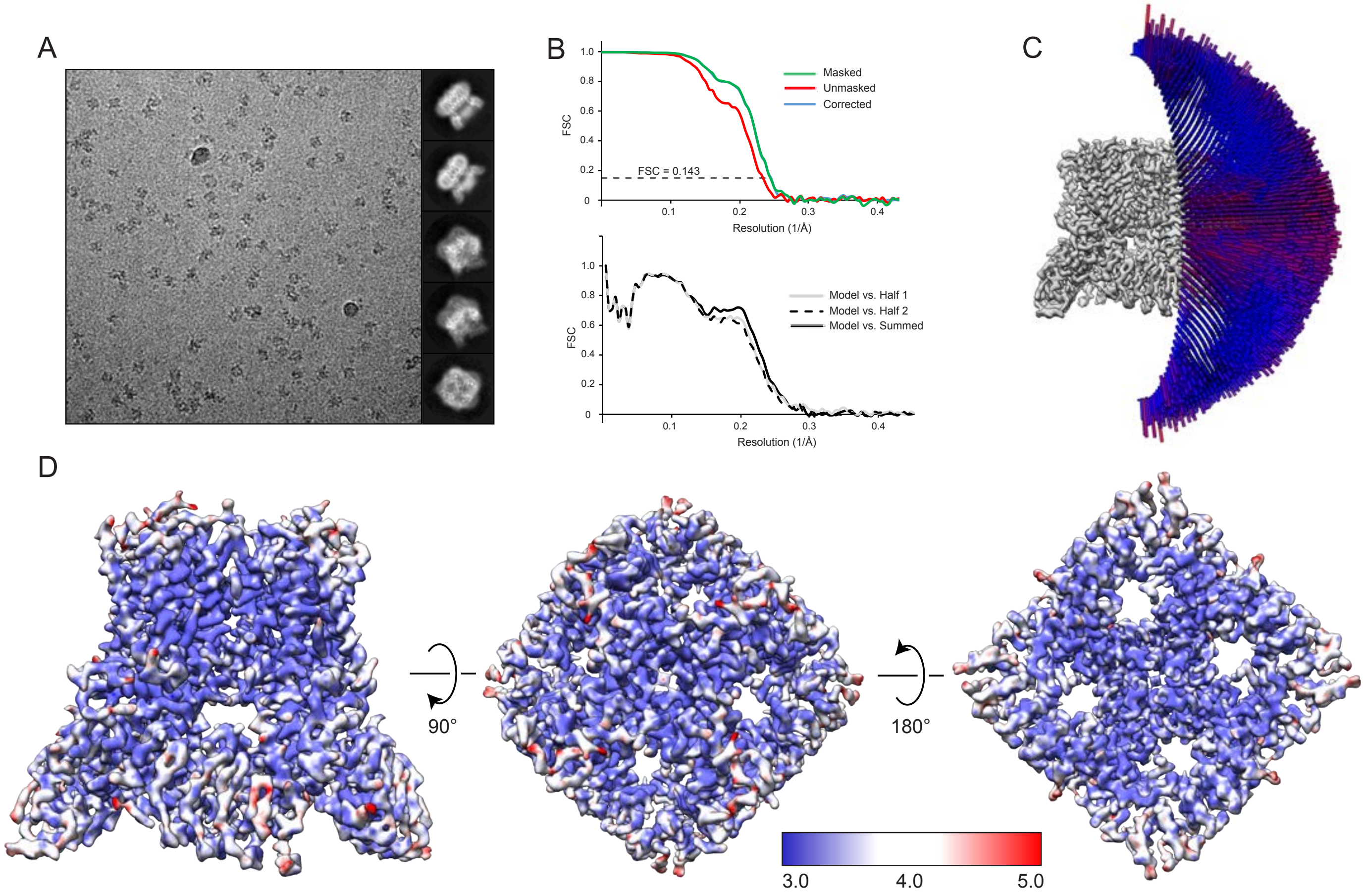

Supplementary Figure 1. Lipid-bound TRPV5 structure in detergent. (**A**) A representative micrograph and 2D classes for lipid-bound TRPV5. (**B**) FSC curves for the masked (green), unmasked (red) and corrected (blue) maps (top). The dotted line indicates an FSC of 0.143. FSC curves comparing the model to density maps (bottom). (**C**) The angular distribution of views is shown for the C4 refined map. (**D**) Local resolution is shown for lipid-bound TRPV5 in three different orientations on a scale of 3.0 Å (blue) to 5.0 Å (red).

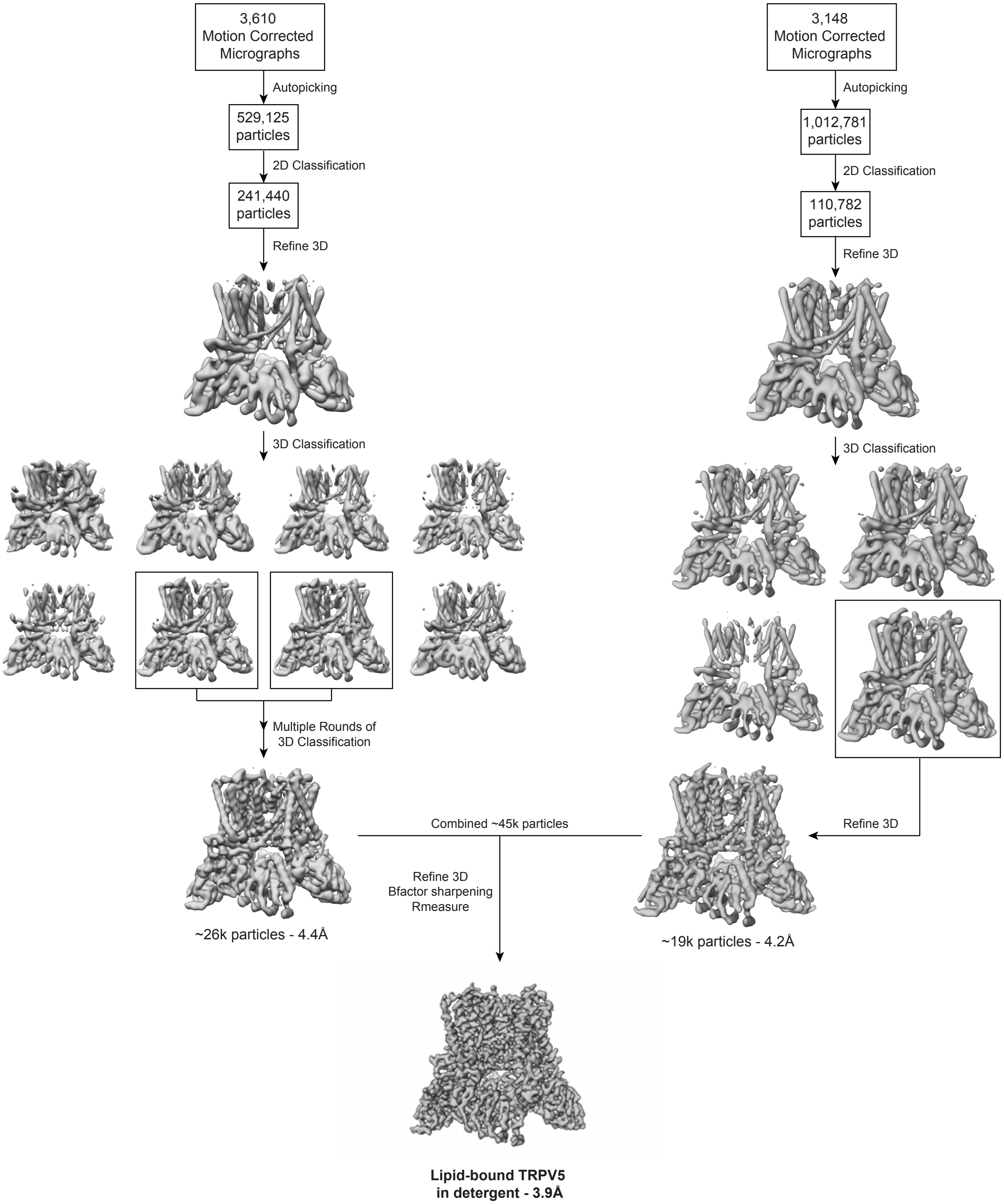

Supplementary Figure 2. Lipid-bound TRPV5 data processing. Workflow for solving the lipid-bound TRPV5 structure to 3.9Å in detergent.

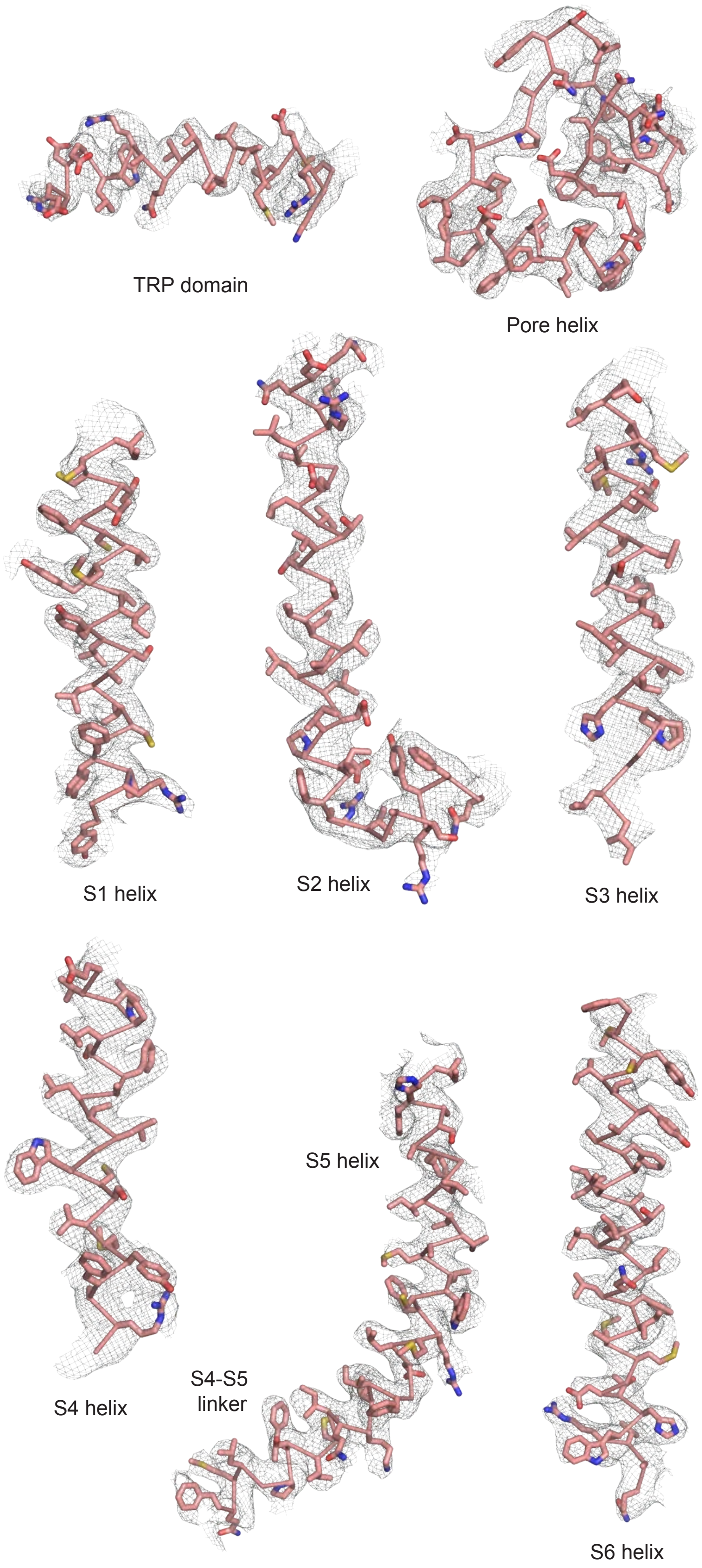

Supplementary Figure 3. Lipid-bound TRPV5 model. Various helices of the lipid-bound TRPV5 model (pink ribbon) overlaid with the lipid-bound TRPV5 density map (mesh). Residues are shown as sticks to illustrate the accuracy of the model. All helices shown are within the 3.0 - 4.0 Å region of the structure.

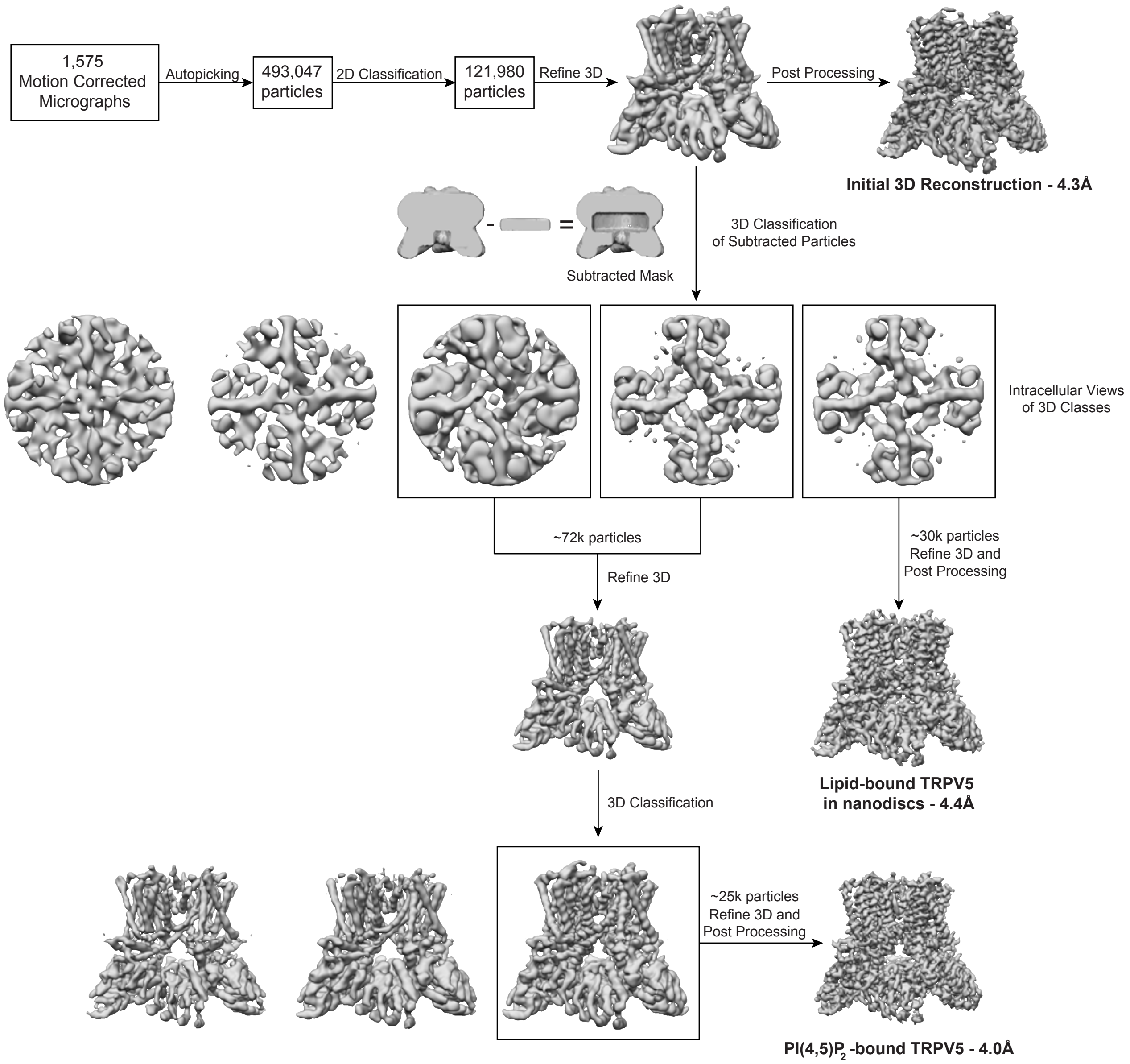

Supplementary Figure 4. TRPV5 nanodisc data processing. The workflow used to reconstruct the PI(4,5)P_2-bound structure of TRPV5 at 4.0 Å resolution and the lipid-bound TRPV5 structure at 4.4 Å resolution in nanodiscs. The TRPV5 data in nanodiscs was initially collected and refined at 4.3 Å resolution (Initial 3D Reconstruction). Using multiple rounds of focused 3D classification, a 4.0 Å structure of PI(4,5)P_2-bound TRPV5, and a 4.4Å structure of lipid-bound TRPV5 were obtained.

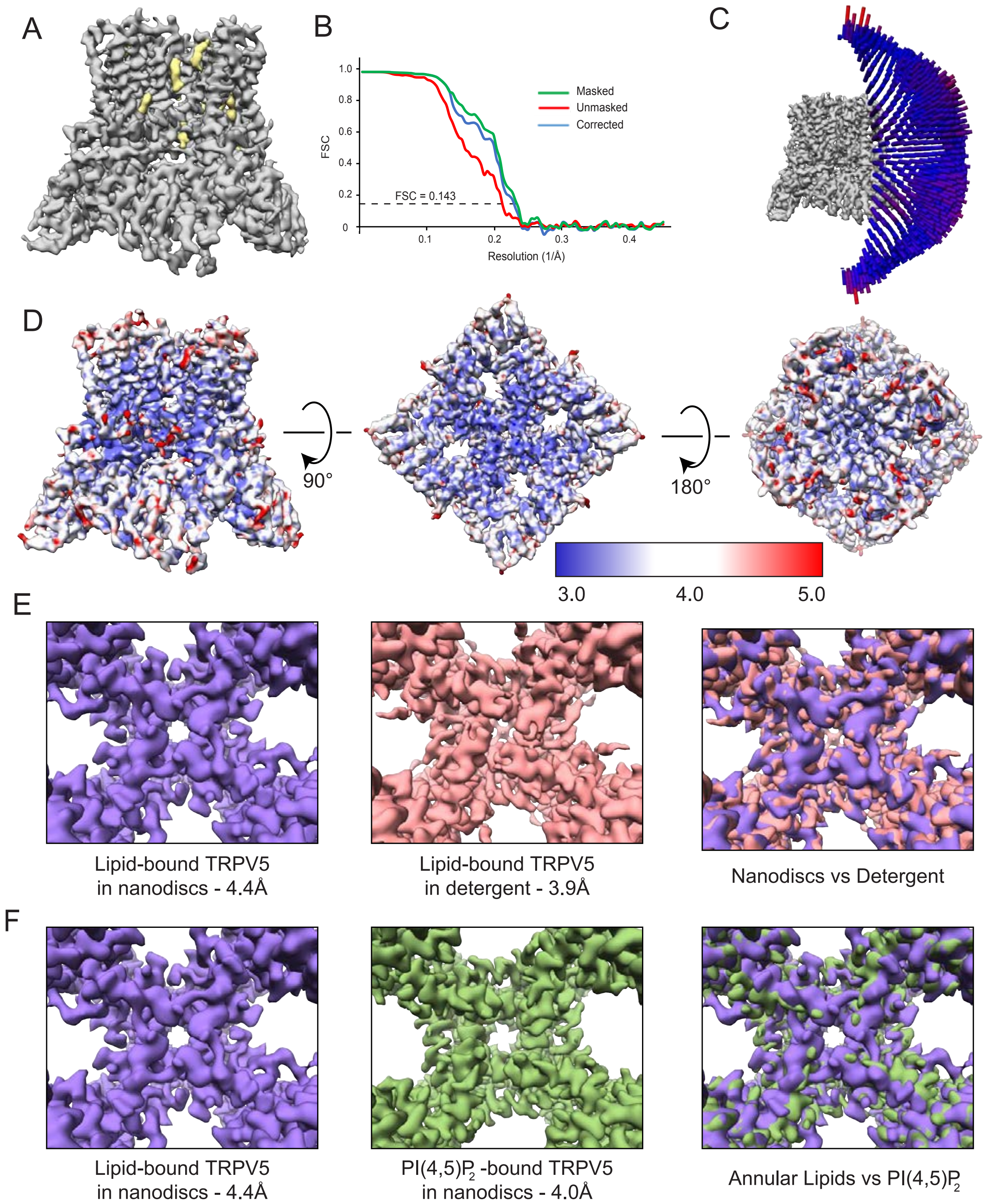

Supplementary Figure 5. Lipid-bound TRPV5 structure in nanodiscs. (**A**) Density map of lipid-bound TRPV5 at 4.4 Å resolution in nanodiscs. Density for TRPV5 is shown in grey and the densities attributed to annular lipids are shown in khaki. (**B**) FSC curves for the masked (green), unmasked (red) and corrected (blue) maps. The dotted line indicates an FSC of 0.143. (**C**) The angular distribution of views is shown for the C4 refined map. (**D**) Local resolution is shown for lipid-bound TRPV5 in nanodiscs in three different orientations on a scale of 3.0 Å (blue) to 5.0 Å (red). (**E**) Comparison between lipid-bound TRPV5 structures in nanodiscs and detergent at the lower gate region. (**F**) Comparison between lipid-bound and PI(4,5)P_2-bound TRPV5 structures in nanodiscs at the lower gate region.

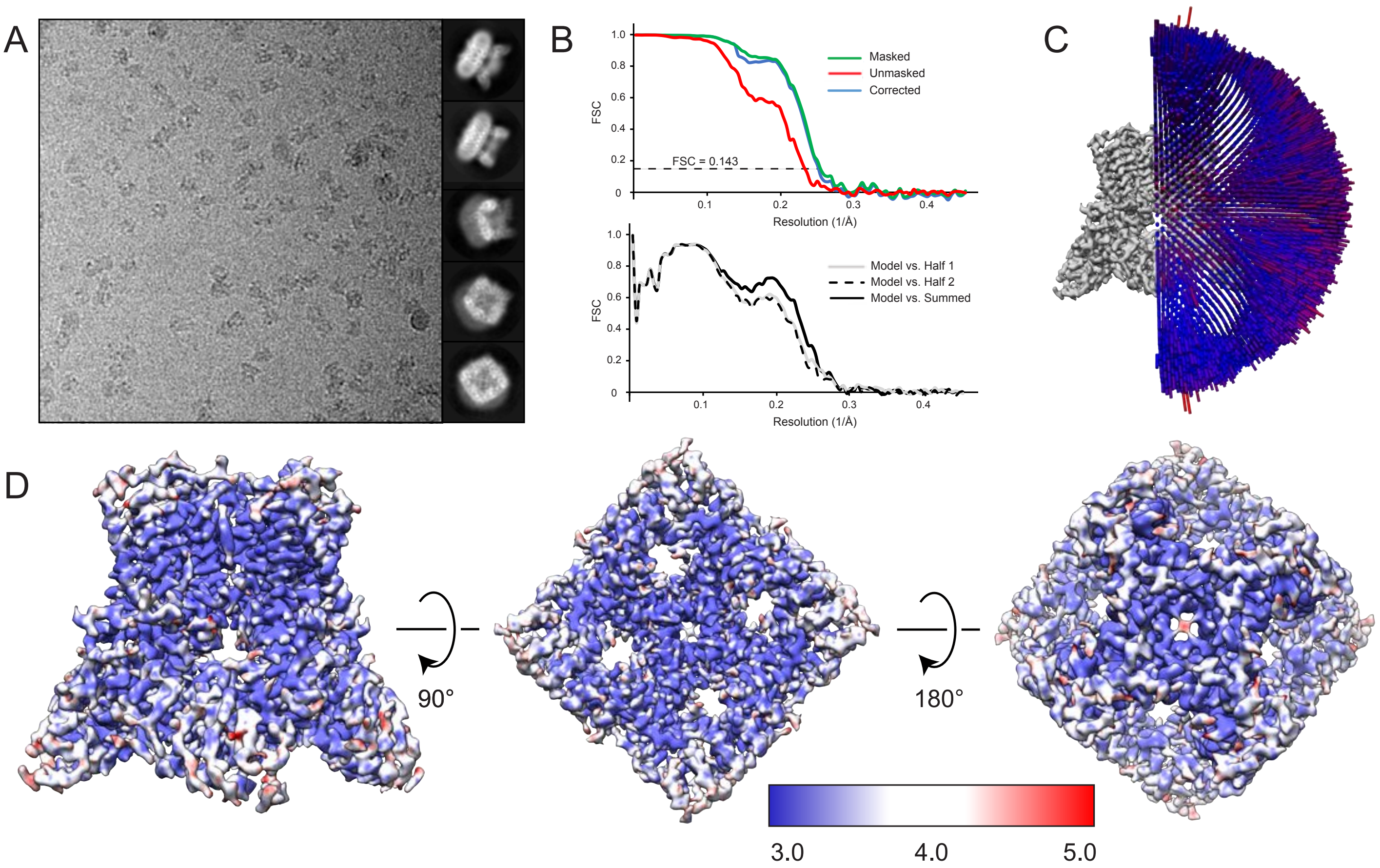

Supplementary Figure 6. PI(4,5)P$_2$-bound TRPV5 structure in nanodiscs. (**A**) A representative micrograph and 2D classes for PI(4,5)P$_2$-bound TRPV5 in nanodiscs. (**B**) FSC curves for the masked (green), unmasked (red) and corrected (blue) maps (top). The dotted line indicates an FSC of 0.143. FSC curves comparing the model to density maps (bottom). (**C**) The angular distribution of views is shown for the C4 refined map. (**D**) Local resolution is shown for PI(4,5)P$_2$-bound TRPV5 in three different orientations on a scale of 3.0Å (blue) to 5.0Å (red).

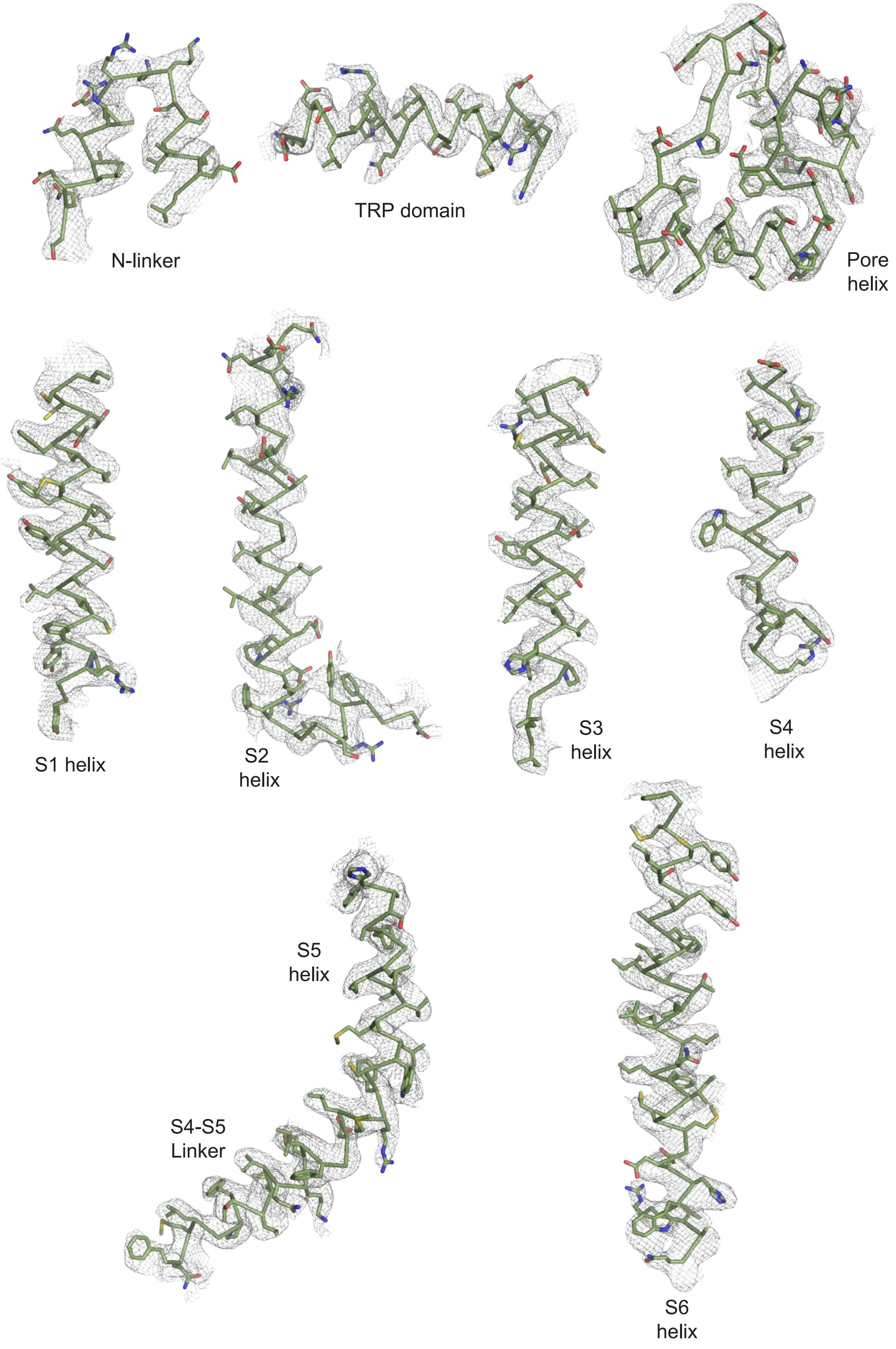

Supplementary Figure 7. PI(4,5)P_2-bound TRPV5 model. Various helices of the PI(4,5)P_2-bound TRPV5 model (green ribbon) overlaid with the PI(4,5)P_2-bound TRPV5 density map (mesh). Residues are shown as sticks to illustrate the accuracy of the model. All helices shown are within the 3.0 - 4.0 Å region of the structure.

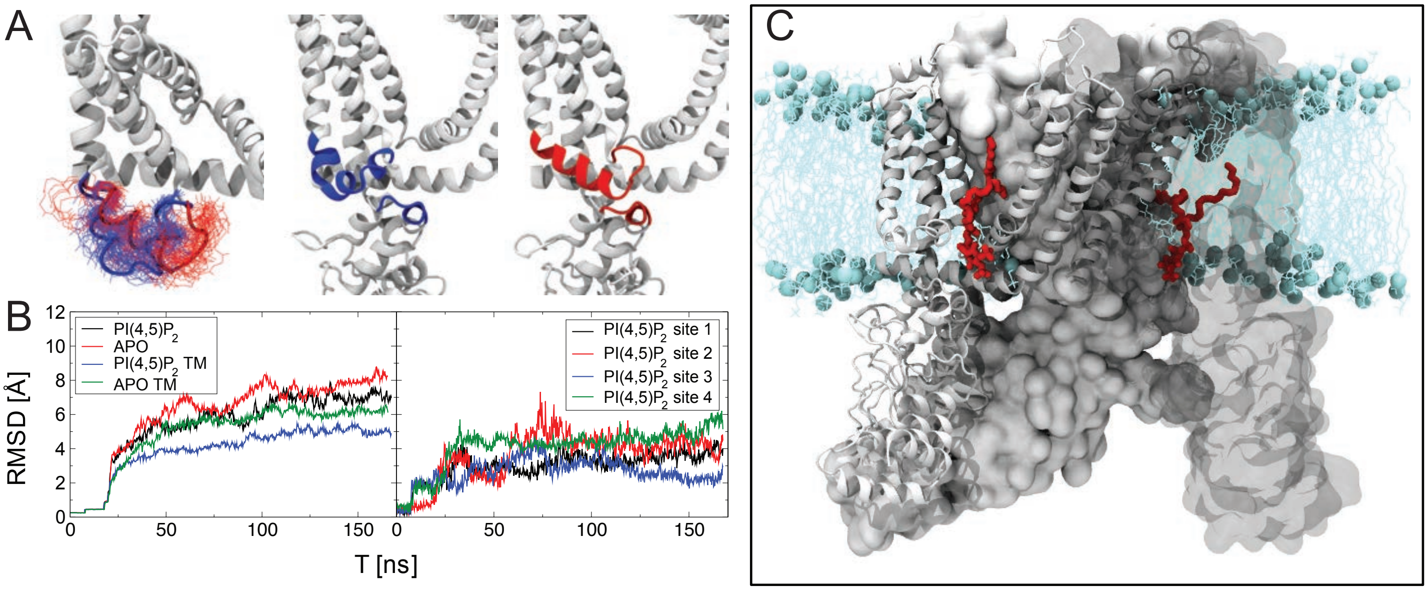

Supplementary Figure 8. TRPV6 MD model and possible binding sites for PI(4,5)P$_2$. (**A**) Possible loop trajectories are seen between the S2 and S3 helices in two major conformations denoted in red and blue (left). Energy refined model for conformation 1 (center). Energy refined model for conformation 2 (right). (**B**) Plot of changes in RMSD as a function of time between PI(4,5)P$_2$ and apo structures of the model (left). Plot of changes in RMSD as a function of time between four PI(4,5)P$_2$ binding sites (right). (**C**) MD model representation of PI(4,5)P$_2$ bound to TRPV6. Depicted in grey is TRPV6, in red is PI(4,5)P$_2$, and in cyan are phospholipids.

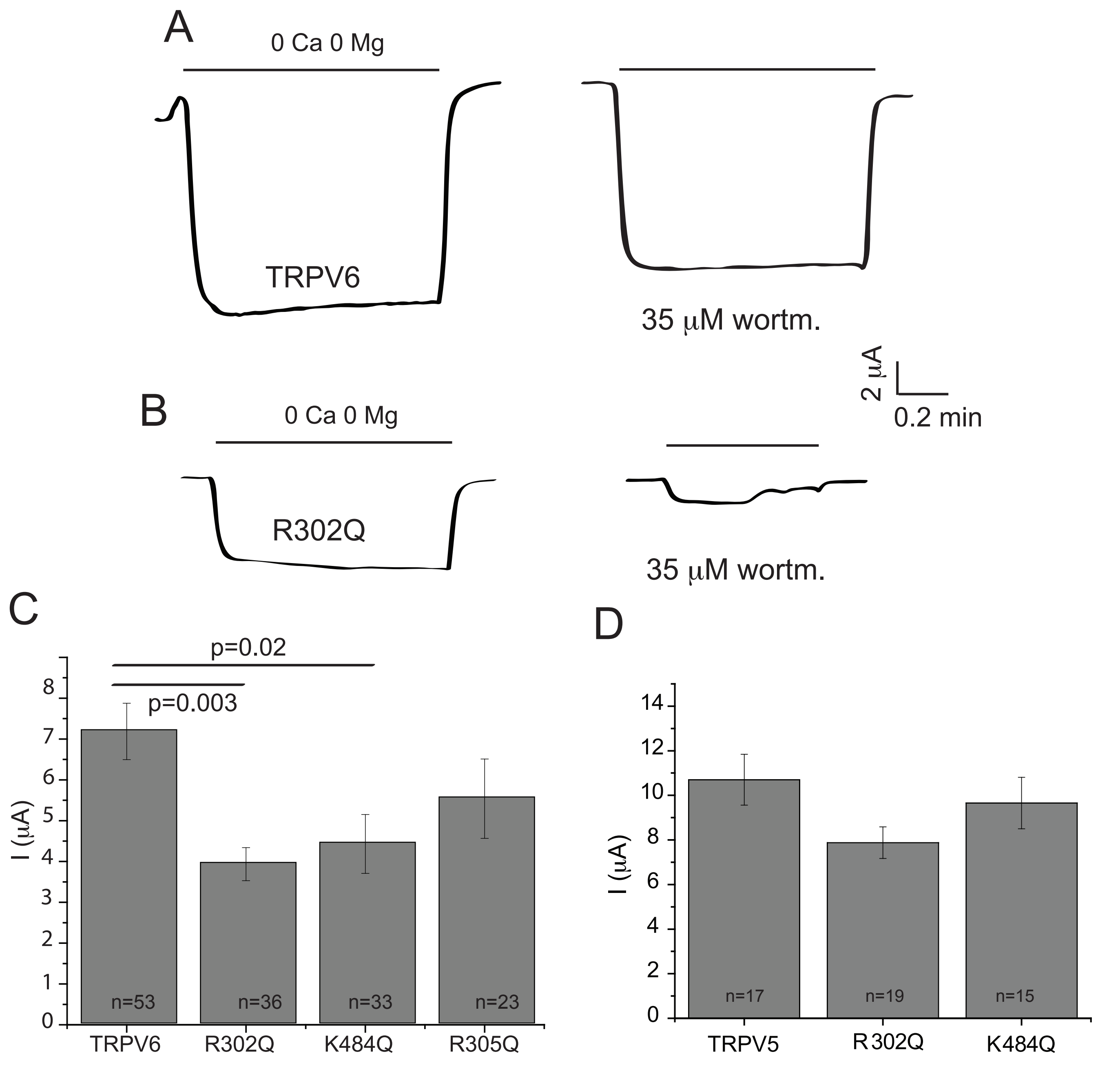

Supplementary Figure 9. Representative current traces and raw current amplitudes for TRPV6, TRPV5 and their mutants. Two electrode voltage clamp measurements were performed as described in the methods section on Xenopus oocytes expressing TRPV6, TRPV5 or their mutants. (**A**) and (**B**) show representative traces for TRPV6 and its R302Q mutant, monovalent currents were induced by exposing oocytes to a Ca^{2+} and Mg^{2+} free solution. After currents were measured, oocytes were incubated with 35 μM wortmannin for 1hr and currents were measured again in the same oocytes (right panels). Summary data for these mutants as well as for other TRPV6 and TRPV5 mutants is shown in Figure 2E and 2F (**C**) Raw current amplitudes of TRPV6 and its mutants. P-values for significance values are shown after rounding to the first non-zero digit (analysis of variance). (**D**) Raw current amplitudes for TRPV5 and its mutants, p=0.11 and 0.75 for the difference between TRPV5 and R302Q and K484Q respectively (analysis of variance). The sample size (*n*) indicates the number of individual oocytes tested from at least two different oocyte preparations. Error bars represent +/- S.E.M.

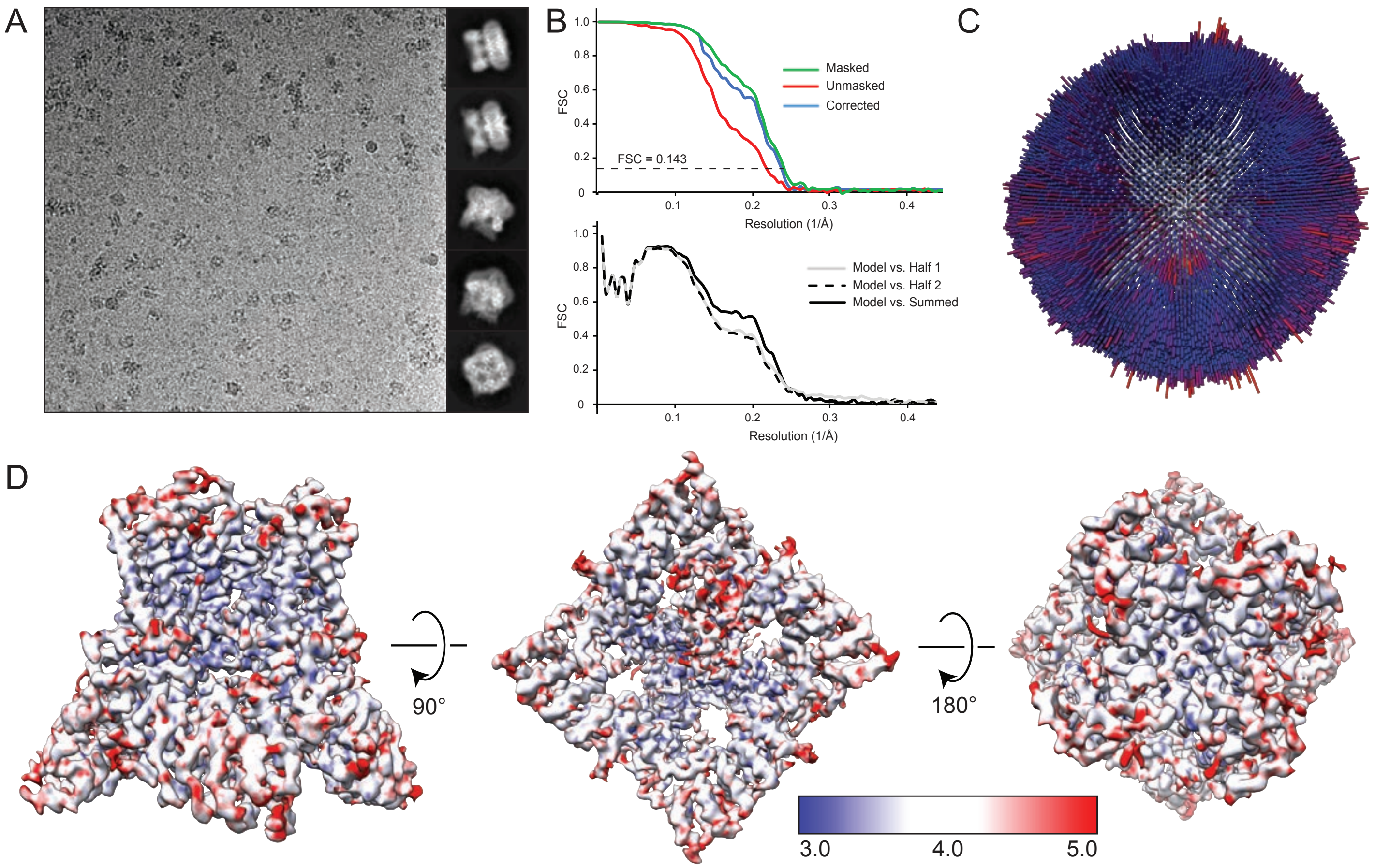

Supplementary Figure 10. CaM-bound TRPV5 structure. (**A**) A representative micrograph and 2D classes for CaM-bound TRPV5. (**B**) FSC curves for the masked (green), unmasked (red) and corrected (blue) maps (top).The dotted line indicates an FSC of 0.143. FSC curves comparing the model to density maps (bottom). (**C**) The angular distribution of views is shown for the C1 refined map. (**D**) Local resolution is shown for CaM-bound TRPV5 in three different orientations on a scale of 3.0Å (blue) to 5.0Å (red).

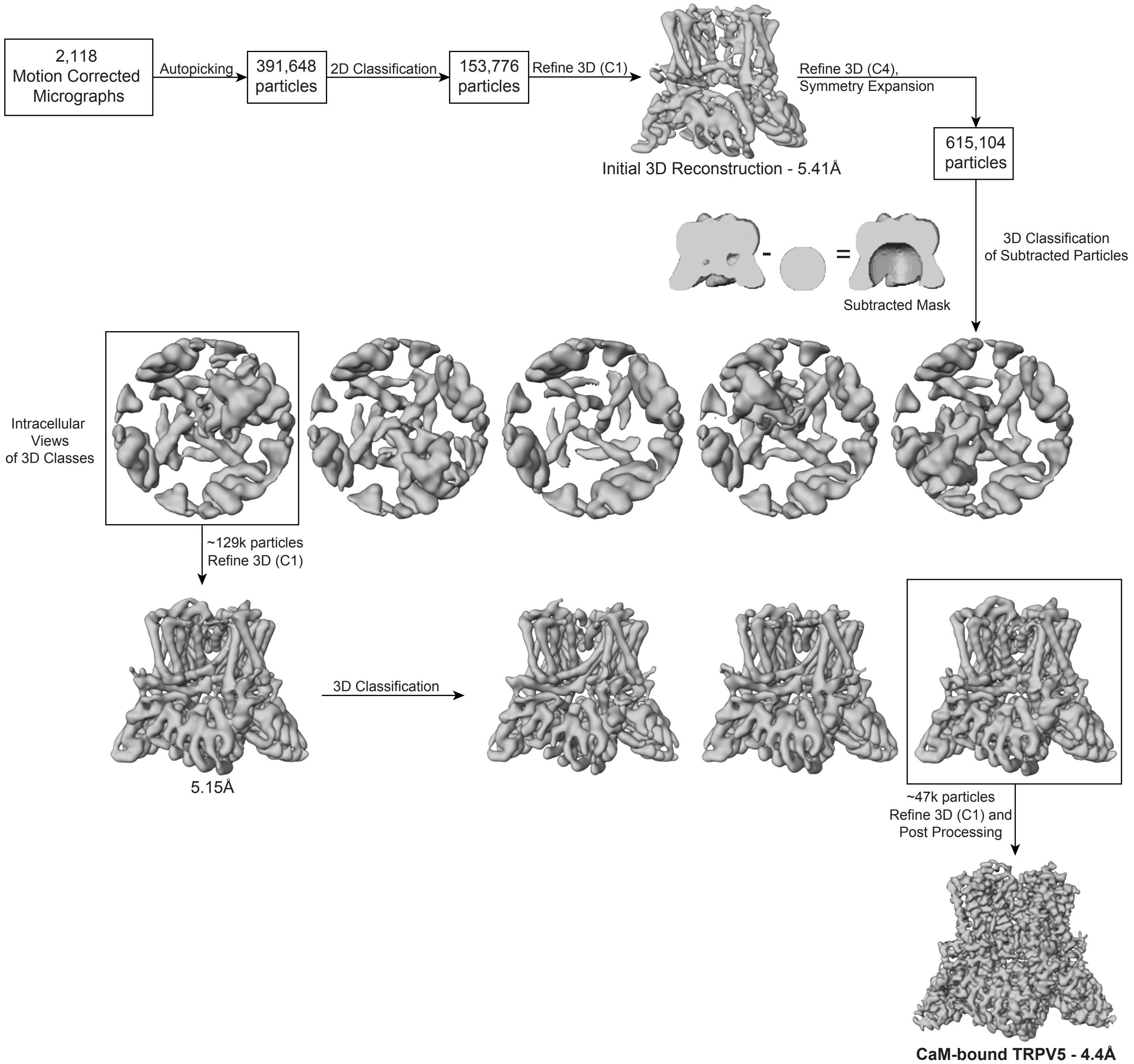

Supplementary Figure 11. CaM-bound TRPV5 data processing. The workflow used to solve the C1 symmetric CaM-bound TRPV5 structure to 4.4Å.

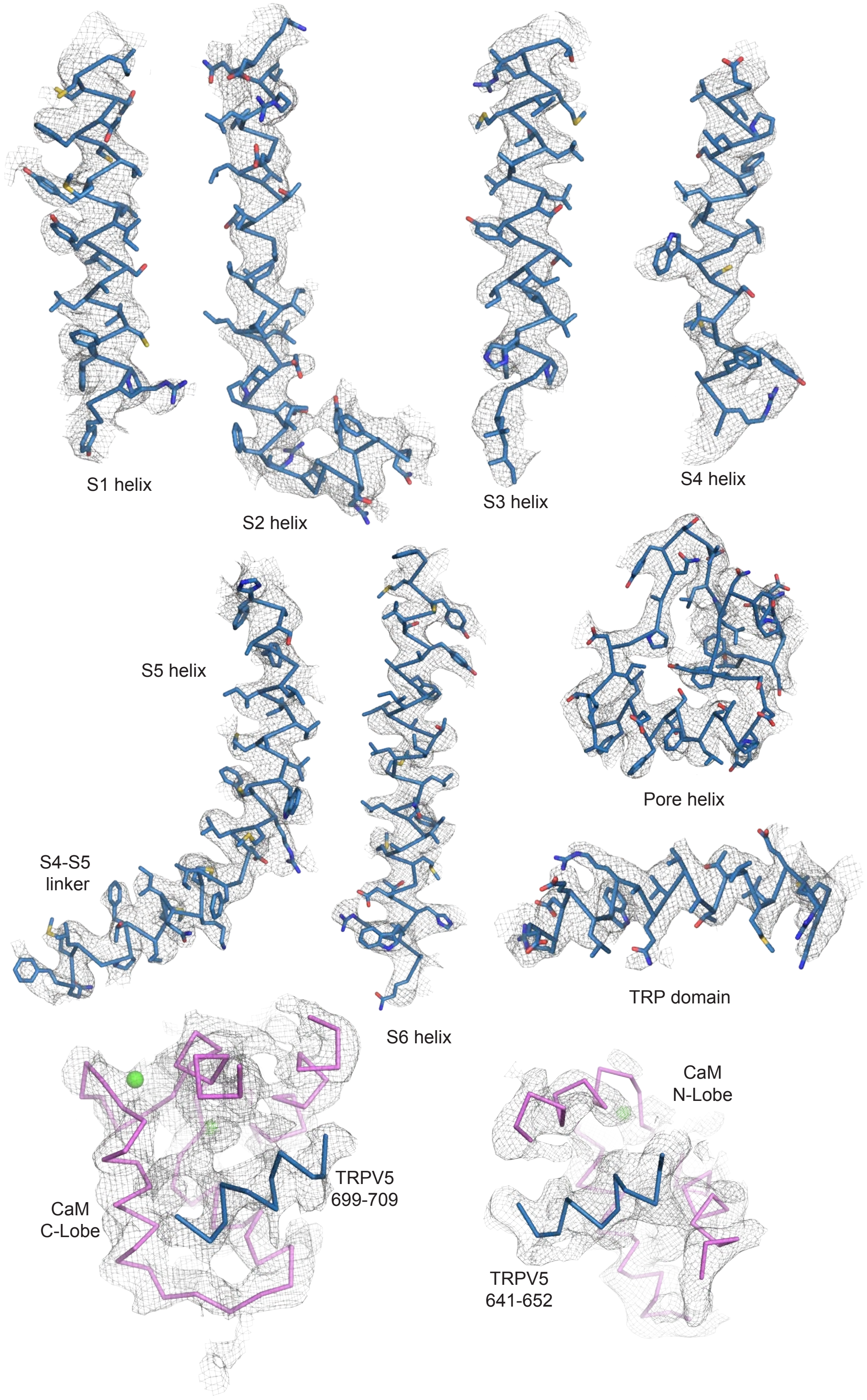

Supplementary Figure 12. CaM-bound TRPV5 model. Various helices of the CaM-bound TRPV5 model (ribbon, blue for TRPV5 and hot pink for CaM) overlaid with the CaM-bound TRPV5 density map (mesh). Residues are shown as sticks to illustrate the accuracy of the model. All TMD helices shown are within the 3.0 - 4.0 Å region of the structure. CaM and TRPV5 C-terminal binding partners shown only as ribbons.

Supplementary Table 1. Cryo-EM data collection and model statistics

	Lipid-Bound TRPV5 in Detergent (EMB-7965, PDB 6DMR)	PI(4,5)P_2-Bound TRPV5 in Nanodiscs (EMB-7966, PDB 6DMU)	CaM-Bound TRPV5 in Detergent (EMB-7967 , PDB 6DMW)
**Data collection and processing**			
Magnification	~45,500	~45,500	~45,500
Voltage (kV)	300	300	300
Defocus range (μm)	1.0-2.5	1.0-2.5	1.25-2.5
Pixel size (Å)	1.1	1.1	1.1
Symmetry imposed	C4	C4	C1
Initial particle images (no.)	>1.5 million	493,047	391,648
Final particle images (no.)	45,354	25,538	47,484
Map resolution (Å)	3.9	4.0	4.4
FSC threshold	0.143	0.143	0.143
Map resolution range (Å)	3.0-5.0	3.0-5.0	3.0-5.0
**Refinement**			
Model resolution cut-off (Å)	3.9	4.0	4.4
FSC threshold	0.143	0.143	0.143
Map sharpening *B* factor ($Å^2$)	-174	-150	-200
Model composition			
Nonhydrogen atoms	0	0	3
Protein residues	2424	2412	2562
Ligands	0	4	0
R.m.s. deviations			
Bond lengths (Å)	0.006	0.006	0.005
Bond angles (°)	1.18	1.28	0.92
Validation			
MolProbity score	1.44	1.29	1.64
Clashscore	3.57	1.71	6.63
Poor rotamers (%)	0.00	0.19	0.18
Ramachandran plot			
Favored (%)	95.76	94.69	95.98
Allowed (%)	4.24	5.31	4.02
Disallowed (%)	0.00	0.00	0.00
