# Supplementary material for: Structural insights on TRPV5 gating by endogenous modulators
Source: Nat Commun. 2018 Oct 10;9:4198. doi: 10.1038/s41467-018-06753-6 (PMC6179994; doi:10.1038/s41467-018-06753-6)
Supplement: Supplementary file 3 — Description of Additional Supplementary Files [file 41467_2018_6753_MOESM3_ESM.pdf]

Description of Additional Supplementary Files

File Name: Supplementary Movie 1
Description: TRPV5 activation by PI(4,5)P2. Morph between lipid-bound TRPV5 and PI(4,5)P2- bound TRPV5 models, depicted as cartoons. Zoomed out view shows Trp583 as sticks. Zoomed in view shows Arg302, Lys484, Trp583, Arg 584, Gln587, Glu588 and PI(4,5)P2 as sticks
