# Supplementary material for: Structural insights on TRPV5 gating by endogenous modulators
Source: Nat Commun. 2018 Oct 10;9:4198. doi: 10.1038/s41467-018-06753-6 (PMC6179994; doi:10.1038/s41467-018-06753-6)
Supplement: Supplementary file 5 — Reporting Summary [file 41467_2018_6753_MOESM5_ESM.pdf]

nature research

Corresponding author(s): Vera Y. Moiseenkova-Bell

Reporting Summary

Nature Research wishes to improve the reproducibility of the work that we publish. This form provides structure for consistency and transparency in reporting. For further information on Nature Research policies, see Authors & Referees and the Editorial Policy Checklist.

Statistical parameters

When statistical analyses are reported, confirm that the following items are present in the relevant location (e.g. figure legend, table legend, main text, or Methods section).

n/a	Confirmed	
☐	☒	The exact sample size (*n*) for each experimental group/condition, given as a discrete number and unit of measurement
☐	☒	An indication of whether measurements were taken from distinct samples or whether the same sample was measured repeatedly
☐	☒	The statistical test(s) used AND whether they are one- or two-sided<br>*Only common tests should be described solely by name; describe more complex techniques in the Methods section.*
☒	☐	A description of all covariates tested
☒	☐	A description of any assumptions or corrections, such as tests of normality and adjustment for multiple comparisons
☐	☒	A full description of the statistics including central tendency (e.g. means) or other basic estimates (e.g. regression coefficient) AND variation (e.g. standard deviation) or associated estimates of uncertainty (e.g. confidence intervals)
☒	☐	For null hypothesis testing, the test statistic (e.g. *F*, *t*, *r*) with confidence intervals, effect sizes, degrees of freedom and *P* value noted<br>*Give P values as exact values whenever suitable.*
☒	☐	For Bayesian analysis, information on the choice of priors and Markov chain Monte Carlo settings
☒	☐	For hierarchical and complex designs, identification of the appropriate level for tests and full reporting of outcomes
☒	☐	Estimates of effect sizes (e.g. Cohen's *d*, Pearson's *r*), indicating how they were calculated
☐	☒	Clearly defined error bars<br>*State explicitly what error bars represent (e.g. SD, SE, CI)*

Our web collection on statistics for biologists may be useful.

Software and code

Policy information about availability of computer code

Data collection	Leginon
Data analysis	Relion, MotionCor2, GCTF, RESMAP, COOT, PHENIX, rmeasure, Bfactor, ROSETTA, HOLE, AUTODOCK, NAMD 2.10

For manuscripts utilizing custom algorithms or software that are central to the research but not yet described in published literature, software must be made available to editors/reviewers upon request. We strongly encourage code deposition in a community repository (e.g. GitHub). See the Nature Research guidelines for submitting code & software for further information.

Data

Policy information about availability of data

All manuscripts must include a data availability statement. This statement should provide the following information, where applicable:

- Accession codes, unique identifiers, or web links for publicly available datasets
- A list of figures that have associated raw data
- A description of any restrictions on data availability

The cryo-EM density maps and atomic coordinates of all structures presented in the text are deposited into the Electron Microscopy Data Bank and Protein Data Bank under the following access codes: Lipid-Bound TRPV5 in detergent (EMB-7965, PDB 6DMR); PI(4,5)P2-Bound TRPV5 in nanodiscs (EMB-7966, PDB 6DMU); CaM-Bound TRPV5 in detergent (EMB-7967, PDB 6DMW). All data is available from the corresponding author upon reasonable request.

Field-specific reporting

Please select the best fit for your research. If you are not sure, read the appropriate sections before making your selection.

☒ Life sciences ☐ Behavioural & social sciences ☐ Ecological, evolutionary & environmental sciences

For a reference copy of the document with all sections, see nature.com/authors/policies/ReportingSummary-flat.pdf

Life sciences study design

All studies must disclose on these points even when the disclosure is negative.

Sample size	Data was analyzed by one-way ANOVA with Bonferroni post hoc comparison
Data exclusions	Data were not excluded from analysis.
Replication	Replication was successful.
Randomization	N/A
Blinding	N/A

Reporting for specific materials, systems and methods

Materials & experimental systems

n/a	Involved in the study	
☒	☐	Unique biological materials
☐	☒	Antibodies
☐	☒	Eukaryotic cell lines
☒	☐	Palaeontology
☐	☒	Animals and other organisms
☒	☐	Human research participants

Methods

n/a	Involved in the study	
☒	☐	ChIP-seq
☒	☐	Flow cytometry
☒	☐	MRI-based neuroimaging

Antibodies

Antibodies used	We used 1D4 antibody that has been produced using hybridoma cell line in CWRU and University of Pennsylvania core facilities
Validation	Antibodies were validated in-house.

Eukaryotic cell lines

Policy information about cell lines

Cell line source(s)	BJ5457 S. cerevisiae strain from ATCC, HEK293
Authentication	this is an yeast cell line, Saccharomyces cerevisiae Meyen ex E.C. Hansen (ATCC® 208282™)
Mycoplasma contamination	None
Commonly misidentified lines (See ICLAC register)	N/A

Animals and other organisms

Policy information about studies involving animals; ARRIVE guidelines recommended for reporting animal research

Laboratory animals	Xenopus oocytes

Wild animals	N/A
Field-collected samples	N/A
