# Supplementary material for: Structural insights on TRPV5 gating by endogenous modulators
Source: Nat Commun. 2018 Oct 10;9:4198. doi: 10.1038/s41467-018-06753-6 (PMC6179994; doi:10.1038/s41467-018-06753-6)
Supplement: Supplementary file 2 — Peer Review File [file 41467_2018_6753_MOESM2_ESM.pdf]

Reviewers' comments:

Reviewer #1 (Remarks to the Author):

In the article entitled Structural insights on TRPV5 gating by endogenous modulators the authors present two structures of the TRPV5 channel obtained by cryo-EM and single particle analysis. TRPV5 channels play an important role in Ca2+ homeostasis and enable uptake of Ca2+ in the kidney.

The authors were able to achieve a global resolution of 3.9 Å in the presence of the lipid modulator PI(4,5)P2. While the lipid itself could not be identified, the authors describe binding of four (7) annular lipids to the channel. They suggest that these lipids are important for the structural stability and suggest that the binding sites should be analysed as drugable areas. Earlier this year the authors have solved and published a similar structure (Hughes et al. NSMB. 2018) of TRPV5 at 4.8Å resolution in the presence of its inhibitor econazole. Aside from the structural/biological interpretation the improved density enabled better model building. For the second structure the authors have isolated and imaged the complex of TRPV5 with calmodulin (CaM). This complex accounts for the state of calcium mediated inactivation. The overall resolution of this map is, however, relatively low with 6.2Å. Therefore, the position and orientation of the single CaM that binds to the tetrameric complex could be identified but the overall biological interpretation is very limited.
Clearly the second structure is potentially even more interesting as it displays the regulation of an important membrane protein.

While this work is of potentially great interest to the community I have several major concerns that need to be addressed thoroughly before publication should be considered.

Even though both densities display high resolution features (at different levels) in some areas, they both require higher-resolution to be really impactful. The first structure is a rather incremental improvement as the authors were not able to identify the modulator PI(4,5)P2. The second density shows very strong potential but I am afraid has been submitted prematurely. Unfortunately, for both datasets the interpretation that the authors provide is therefore, very hypothetical and not concrete enough.

General remarks:

I think that the CaM structure has great potential but needs to be reprocessed or requires more data – most likely both. However, in general I miss how the two presented structures intertwine into a single comprehensive story. This is of course due to the structural details that are missing. The manuscript would be much stronger if only the CaM structure, at improved resolution – but not necessarily 3.5Å, was presented and some biological mechanisms would have been drawn from this. IN general 4-3.5Å structures of symmetrical alpha-helical TRPV channels are nowadays at the lower end of the resolution range.
While I tend to believe their biological interpretation, the authors seem to be prone to over-interpretation of their data (as discussed below), which questions the entire quality of the study. The manuscript should be tightened and reworded (see minor comments).

Major technical concerns:

For the CaM-bound structure the authors initially applied C1 symmetry and afterwards C4 symmetry to improve the resolution. Not surprisingly the symmetrisation did the job and increased the resolution levels significantly. However, this must not be done when understanding an asymmetric or pseudo-symmetric particle. Logically the symmetrisation abolishes every information about the asymmetry, which is the important aspect when analysing binding of a single protein to a four-fold particle. Therefore, this entire segment and the interpretation should

be left out. The entire procedure can only serve as a demonstration that the data was in general good enough to obtain 4.9Å, or better in local areas. Therefore, it is also not surprising, as conformational changes are subtle, that this refinement appears identical to the first PI(4,5)P2 bound map.

When reading the methods, I am astonished that the authors have not attempted to utilize some sort of symmetry expansion, as is well established in Frealign, cisTEM and Relion to tackle this problem and obtain higher resolution. Also, from the methods it is not obvious, whether the authors have attempted to perform local masking and classification to improve the quality of the map. Both approaches are standard procedures in the field and need to be considered! Most likely, if applied correctly, they will lead to a much better volume!
For the CaM-bound structure the authors mention accurate side chain information which is obviously a large over-interpretation (as for example: "In both models, there are three residues that are involved in pore constriction: Asp542, Ile575 and Trp583 (Fig. 4A- B).").
At this resolution levels one can accurately position alpha helices.

The fact that the authors claim to have identified clear density for two calcium ions at this resolution is such a dramatic over interpretation that it puts the entire manuscript at question!

With this in mind I have serious doubts about the strong interpretation of the extra densities that were identified as annular lipids bound to the first structure by the authors.

Minor concerns:

The figures and color codes are too dark and do not have sufficient contrast.
Especially in Figure 2 C the potential Ca ions are impossible to spot.

The manuscript should be significantly shortened and many phrases should be left out entirely:

In spite of the high quality of this map

without applied symmetry (referred to as C1 symmetry)

C1 symmetry is defined and does need further explanation.

the C1 symmetry map was not high enough resolution to refine sidechain placement

Logically, it is secondary structure resolution

Nevertheless, at these resolutions we cannot definitively say one way or the other as a wide variety of lipids can be docked into the densities

Obviously, but the authors spent a lot of time on discussing what could be – they need better data.

The sentences in regards to the nanodiscs are also quite confusing and should be removed. It has been demonstrated before that annular lipids can be co-purified with detergent solubilized membrane proteins.

Reviewer #2 (Remarks to the Author):

TRPV5 is a calcium-selective channel highly expressed in the apical membrane of certain kidney epithelial cells and plays an important role in calcium reabsorption in the kidney. Previous functional studies have shown that TRPV5 activity is tightly regulated by endogenous modulators, including PI(4,5)P2 and calmodulin (CaM), which stimulate and inhibit TRPV5 activity, respectively. A better understanding of the molecular mechanisms of both forms of regulation is of intrinsic importance and may lead to new approaches to target these channels as drug targets.
In this interesting study, the authors aimed to elucidate the structural basis of PI(4,5)P2 and CaM regulation and obtained cryo-EM structures of TRPV5 in the presence of diC8 PI(4,5)P2, a short chain PI(4,5)P2, and calcium-bound CaM. The quality of the structural data is good, and the authors were rightly cautious when called for in their modeling and interpretation. Despite the limited resolution, the CaM-bound structure convincingly reveals that one CaM binds to each channel and inhibits the channel by directly blocking the inner pore. Although many channels are regulated by CaM and many structures of ion channel/CaM complexes have been reported, to my knowledge, this is the first structure showing a direct pore block by CaM. This new regulatory mechanism by CaM is a highlight of this work.
The structures obtained in the presence of diC8 PI(4,5)P2 and CaM show multiple lipids, consistent with the observation of lipid modulation of TRPV5 activity. However, the authors were unable to definitely identify the PI(4,5)P2 binding site. Indeed, the high similarity between the lipid-bound and CaM-bound structures suggest that diC8 PI(4,5)P2 is not present in the lipid-bound structure. This shortcoming significantly lessens the impact of this study and should be addressed by using alternative approaches such as nanodisc reconstitution. Without identifying the PI(4,5)P2 binding site and elucidating how PI(4,5)P2 enhances channel activity, this work seems incomplete and unsatisfying.
Minor points:
1. In Figure 4C, the authors assign two putative calcium ions in the pore, one at the selectivity filter and one at the lower gate, in the lipid-bound structure. However, no calcium was added in Buffer B. What is the free calcium concentration in Buffer B? The authors should explain this experiment and result in more detail.
2. Related to the question above, how confident the authors are that the CaM in the CaM-bound structure is calcified? Although 10 mM calcium was added to the TRPV5-CaM mixture, my understanding is that the complex was finally purified in Buffer B, which has no added calcium and whose free calcium concentration is not stated. Please clarify.
3. W583 and Q587 are identified as key amino acids for CaM C-lobe interaction and CaM inhibition. The authors should provide more information on these amino acids and functionally validate their importance. Are they present in TRPV6? What happens to CaM inhibition if they are mutated, either individually or in combination?
4. It might be interesting and helpful to compare and contrast CaM inhibition of TRPV5 and the TRPV5-CaM structure with CaM modulation of some other TRP channels and CaM-bound structures.

We thank both reviewers for the time and energy taken to review our manuscript. In the revised manuscript, we addressed the reviewers' major concerns which we believe made our manuscript significantly more robust. In addition to the requested changes and new data, we also incorporated functional and computational modeling data from our collaborators on interaction of the closely related TRPV6 with PI(4,5)P_2. Their data, obtained independently from our structure determination, show an essentially identical binding mode of PI(4,5)P_2 to TRPV6, suggesting that this interaction may be conserved between these two closely related epithelial channels.

Our detailed responses are below; for ease of navigation of this document, we have colored the original reviewers' comments in black and colored our responses in blue.

Reviewer #1 (Remarks to the Author):

In the article entitled Structural insights on TRPV5 gating by endogenous modulators the authors present two structures of the TRPV5 channel obtained by cryo-EM and single particle analysis. TRPV5 channels play an important role in Ca2+ homeostasis and enable uptake of Ca2+ in the kidney. The authors were able to achieve a global resolution of 3.9 Å in the presence of the lipid modulator PI(4,5)P2. While the lipid itself could not be identified, the authors describe binding of four (7) annular lipids to the channel. They suggest that these lipids are important for the structural stability and suggest that the binding sites should be analyzed as drugable areas. Earlier this year the authors have solved and published a similar structure (Hughes et al. NSMB. 2018) of TRPV5 at 4.8Å resolution in the presence of its inhibitor econazole. Aside from the structural/biological interpretation the improved density enabled better model building. For the second structure the authors have isolated and imaged the complex of TRPV5 with calmodulin (CaM). This complex accounts for the state of calcium mediated inactivation. The overall resolution of this map is, however, relatively low with 6.2Å. Therefore, the position and orientation of the single CaM that binds to the tetrameric complex could be identified but the overall biological interpretation is very limited. Clearly the second structure is potentially even more interesting as it displays the regulation of an important membrane protein. While this work is of potentially great interest to the community I have several major concerns that need to be addressed thoroughly before publication should be considered. Even though both densities display high resolution features (at different levels) in some areas, they both require higher-resolution to be really impactful. The first structure is a rather incremental improvement as the authors were not able to identify the modulator PI(4,5)P2. The second density shows very strong potential but I am afraid has been submitted prematurely. Unfortunately, for both datasets the interpretation that the authors provide is therefore, very hypothetical and not concrete enough.

We would like to thank Reviewer #1 for very constructive comments. We found that your critiques helped us to improve the manuscript. By collecting additional data sets and utilizing symmetry expansion, local masking and classification as established in Relion, we were able to obtained a PI(4,5)P_2-bound structure at a global resolution of 4.0Å and a CaM-bound TRPV5 structure at a global resolution of 4.4Å in C1 symmetry. At this resolution, we were able to visualize amino acid side chains in our newly determined TRPV5 structures, which allowed us to propose mechanisms of TRPV5 inactivation by CaM as well as to identify the PI(4,5)P_2 binding site. These findings are also supported by functional data provided in this revised manuscript. We show that the wild type TRPV5 is robustly inhibited by Ca^{2+}-bound CaM in excised inside out patches, and the W583L mutation eliminated this effect (Fig.6 F-H). We also included data to support the functional role of the identified PI(4,5)P_2 binding site on both TRPV5 and the closely related TRPV6 (Fig. 2 E-F).

General remarks:

I think that the CaM structure has great potential but needs to be reprocessed or requires more data – most likely both. However, in general I miss how the two presented structures intertwine into a single comprehensive story. This is of course due to the structural details that are missing. The manuscript would be much stronger if only the CaM structure, at improved resolution – but not necessarily 3.5Å, was presented and some biological mechanisms would have been drawn from this. In general, 4-3.5Å structures of symmetrical alpha-helical TRPV channels are nowadays at the lower end of the resolution range. While I tend to believe their biological interpretation, the authors seem to be prone to over-interpretation of their data (as discussed below), which questions the entire quality of the study. The manuscript should be tightened and reworded (see minor comments).

Our laboratory has limited access to a Titan Krios, nevertheless, since we initially submitted this manuscript we have been able to collect two additional data sets. This new data allowed us to obtain a PI(4,5)P_2-bound structure at global resolution of 4.0Å and a CaM-bound TRPV5 structure at a global resolution of 4.4Å in C1 symmetry. At these resolutions, we were able to visualize amino acid side chains in our newly determined TRPV5 structures. We re-wrote the manuscript and incorporated additional supplementary data to show that our TRPV5 structural models are fitted well into our cryo-EM maps.

Major technical concerns:

For the CaM-bound structure the authors initially applied C1 symmetry and afterwards C4 symmetry to improve the resolution. Not surprisingly the symmetrisation did the job and increased the resolution levels significantly. However, this must not be done when understanding an asymmetric or pseudo-symmetric particle. Logically the symmetrisation abolishes every information about the asymmetry, which is the important aspect when analysing binding of a single protein to a four-fold particle. Therefore, this entire segment and the interpretation should be left out. The entire procedure can only serve as a demonstration that the data was in general good enough to obtain 4.9Å, or better in local areas. Therefore, it is also not surprising, as conformational changes are subtle, that this refinement appears identical to the first PI(4,5)P2 bound map.

When reading the methods, I am astonished that the authors have not attempted to utilize some sort of symmetry expansion, as is well established in Frealign, cisTEM and Relion to tackle this problem and obtain higher resolution. Also, from the methods it is not obvious, whether the authors have attempted to perform local masking and classification to improve the quality of the map. Both approaches are standard procedures in the field and need to be considered! Most likely, if applied correctly, they will lead to a much better volume!
For the CaM-bound structure the authors mention accurate side chain information which is obviously a large over-interpretation (as for example: “In both models, there are three residues that are involved in pore constriction: Asp542, Ile575 and Trp583 (Fig. 4A- B).”). At this resolution levels one can accurately position alpha helices.

With our newly collected data, we utilized symmetry expansion, particle subtraction, local masking and classification, as established in Relion, to improve our CaM-bound structure to 4.4Å refined without applied symmetry. At the current resolution of the CaM-bound structure, we were able to see side chains in our cryo-EM map and build a structural model for the mechanism of TRPV5 channel inactivation by CaM. C4 refinement was used only to establish symmetry operators for symmetry expansion and thereafter no symmetry was applied during classification and refinement, thus all asymmetric information was retained in our final structure. In addition, we performed functional assays to confirm our findings; we show that the W583L mutant of TRPV5 is not inhibited by Ca^{2+}-bound CaM in excised inside out patches (Fig.6 F-H).

The fact that the authors claim to have identified clear density for two calcium ions at this resolution is such a dramatic over interpretation that it puts the entire manuscript at question!

That was a misstatement, the point we were making is that we saw density that fit well to the Ca^{2+}-bound state of the CaM C-lobe, which is why we included those ions in our original model despite the low resolution. We also saw extra density in that map next to the CaM C-lobe which fit well to the TRPV5 C-terminal peptide, which only binds to Ca^{2+}-activated CaM. In our new higher resolution CaM map, both lobes are clearly in Ca^{2+}-activated conformations and we were able to place calcium ions in the three visible EF-hand motifs.

With this in mind I have serious doubts about the strong interpretation of the extra densities that were identified as annular lipids bound to the first structure by the authors.

The TRPV field has had several high-profile papers published highlighting extra density in the transmembrane region which these papers have identified as lipids. Our extra density in this region looks very similar to that published for TRPV1 and TRPV6 in Nature and is clearly visible in the same locations in all three of our structures, including the half maps. Nevertheless, we have removed the lipids from our deposited models.

Minor concerns:

The figures and color codes are too dark and do not have sufficient contrast. Especially in Figure 2 C the potential Ca ions are impossible to spot.

Figures and color codes have been lightened and changed.

The manuscript should be significantly shortened and many phrases should be left out entirely:

In spite of the high quality of this map This phrase has been removed.

without applied symmetry (referred to as C1 symmetry)
C1 symmetry is defined and does need further explanation.
This phrase has been removed.

the C1 symmetry map was not high enough resolution to refine sidechain placement
Logically, it is secondary structure resolution
This phrase has been removed.

Nevertheless, at these resolutions we cannot definitively say one way or the other as a wide variety of lipids can be docked into the densities This phrase has been removed.

Obviously, but the authors spent a lot of time on discussing what could be – they need better data.
In our revised manuscript we now discuss our improved structures.

The sentences in regards to the nanodiscs are also quite confusing and should be removed. It has been demonstrated before that annular lipids can be co-purified with detergent solubilized membrane proteins.
These phrases have been removed.

Unfortunately, we could not shorten the manuscript as we now have additional data that had not been originally included in the manuscript. Nevertheless, we removed the sentences that were outline above.

Reviewer #2 (Remarks to the Author):

TRPV5 is a calcium-selective channel highly expressed in the apical membrane of certain kidney epithelial cells and plays an important role in calcium reabsorption in the kidney. Previous functional studies have shown that TRPV5 activity is tightly regulated by endogenous modulators, including PI(4,5)P2 and calmodulin (CaM), which stimulate and inhibit TRPV5 activity, respectively. A better understanding of the molecular mechanisms of both forms of regulation is of intrinsic importance and may lead to new approaches to target these channels as drug targets.
In this interesting study, the authors aimed to elucidate the structural basis of PI(4,5)P2 and CaM regulation and obtained cryo-EM structures of TRPV5 in the presence of diC8 PI(4,5)P2, a short chain PI(4,5)P2, and calcium-bound CaM. The quality of the structural data is good, and the authors were rightly cautious when called for in their modeling and interpretation. Despite the limited resolution, the CaM-bound structure convincingly reveals that one CaM binds to each channel and inhibits the channel by directly blocking the inner pore. Although many channels are regulated by CaM and many structures of ion channel/CaM complexes have been reported, to my knowledge, this is the first structure showing a direct pore block by CaM. This new regulatory mechanism by CaM is a highlight of this work.
The structures obtained in the presence of diC8 PI(4,5)P2 and CaM show multiple lipids, consistent with the observation of lipid modulation of TRPV5 activity. However, the authors were unable to definitely identify the PI(4,5)P2 binding site. Indeed, the high similarity between the lipid-bound and CaM-bound structures suggest that diC8 PI(4,5)P2 is not present in the lipid-bound structure. This shortcoming significantly lessens the impact of this study and should be addressed by using alternative approaches such as nanodisc reconstitution. Without identifying the PI(4,5)P2 binding site and elucidating how PI(4,5)P2 enhances channel activity, this work seems incomplete and unsatisfying.

Thank you. We appreciate Reviewer #2's valuable critiques. Recently, we were able to obtained PI(4,5)P_2-bound structure at a global resolution of 4.0Å and a CaM-bound TRPV5 structure at a global resolution of 4.4Å in C1 symmetry. At these resolutions, we were able to visualize amino acid side chains in our newly determined TRPV5 structures, which allowed us to propose mechanisms of TRPV5 activation by PI(4,5)P_2 and inactivation by CaM. We used nanodiscs to determine the TRPV5 structure in the presence of PI(4,5)P_2 and unambiguously identified a PI(4,5)P_2 binding site. As explained in response to reviewer 1, we also provide electrophysiological data to support the functional role of the identified PI(4,5)P_2 binding site both for TRPV5 and the closely related TRPV6.

Minor points:

1. In Figure 4C, the authors assign two putative calcium ions in the pore, one at the selectivity filter and one at the lower gate, in the lipid-bound structure. However, no calcium was added in Buffer B. What is the free calcium concentration in Buffer B? The authors should explain this experiment and result in more detail.

We have removed the calcium ions in the pore from our model and references to them in the text.

2. Related to the question above, how confident the authors are that the CaM in the CaM-bound structure is calcified? Although 10 mM calcium was added to the TRPV5-CaM mixture, my understanding is that the complex was finally purified in Buffer B, which has no added calcium and whose free calcium concentration is not stated. Please clarify.

We do not know the exact concentration of the free calcium in our buffer B. However, aqueous buffer solutions with no added calcium usually still contain calcium in the low micromolar range. We have clarified this in the method section of the manuscript. Additionally, CaM has very distinct and well-established calcium-free and calcium-bound configurations. In our new, higher resolution map of CaM-bound TRPV5, both N and C lobes are unambiguously in the calcium-bound configuration.

3. W583 and Q587 are identified as key amino acids for CaM C-lobe interaction and CaM inhibition. The authors should provide more information on these amino acids and functionally validate their importance. Are they present in TRPV6? What happens to CaM inhibition if they are mutated, either individually or in combination?

In our improved CaM-bound TRPV5 structure at global resolution of 4.4Å in C1 symmetry, we were able to determined that W583 of the TRPV5 channel directly interacts with K116 of CaM, which allows CaM to directly block the TRPV5 channel pore. These findings were also supported by functional data provided in this manuscript (Fig. 6 F-H), which showed that mutation of the W583 to L583 completely abolished CaM inhibition of the channel, suggesting that W583 is an essential residue that facilitates this mechanism of CaM inactivation. The same tryptophan residue is located at the bottom of the TRPV6 pore.

4. It might be interesting and helpful to compare and contrast CaM inhibition of TRPV5 and the TRPV5-CaM structure with CaM modulation of some other TRP channels and CaM-bound structures.

We added a section in the text comparing our data to previously published studies of CaM and PI(4,5)P_2 modulation of other TRP channels.

REVIEWERS' COMMENTS:

Reviewer #1 (Remarks to the Author):

The authors have addressed most of my concerns. They have collected more data and were most importantly successful in improving the resolution of the CaM bound state. As I have stated in my original review this structure was particularly unsuitable for publication before - but with the recent improvements it is fine.

Reviewer #2 (Remarks to the Author):

The authors have taken the reviewers' critiques to heart and revised the manuscript extensively. The revised manuscript is much improved, with new and higher-resolution structures, functional data and computational modeling, and provides compelling structural and mechanistic insights into how calmodulin and PI(4,5)P2 regulate TRPV5. This work will be of great interest to searchers in various fields and will certainly have a significant impact on TRP channel studies. The authors have adequately addressed my concerns and questions.
